# Agronomic performance and grain quality assessment of selected maize genotypes in the Black Sea Region of Türkiye

**DOI:** 10.3389/fpls.2026.1880606

**Published:** 2026-08-06

**Authors:** Erkan Özata

**Affiliations:** Department of Field Crops, Black Sea Agricultural Research Institute, Samsun, Türkiye

**Keywords:** genotype evaluation, grain quality, grain yield, mineral composition, protein content, *Zea mays* L.

## Abstract

**Introduction:**

This study assessed the agronomic performance, grain composition, and mineral traits of six maize genotypes grown under Black Sea conditions in Türkiye during the 2020 and 2021 growing seasons.

**Methods:**

The experiment took place in Samsun-Bafra using a randomized complete block design with four replications. Agronomic and morphological traits included days to pollen shedding, plant height, ear height, grain moisture, and grain yield, while grain quality was evaluated through compositional traits (ash, fat, protein, cellulose, and starch) and mineral concentrations (Fe, Cu, Mn, Ca, and Mg). Data were analyzed using linear mixed-effects models, while correlation and principal component analyses were used only as exploratory descriptive tools to visualize general trait relationships and genotype-year tendencies.

**Results:**

Significant effects of genotype and year were observed for days to pollen shedding, plant height, and ear height. Grain moisture was significantly influenced by year and the interaction between genotype and year, whereas grain yield was primarily affected by genotype. Among the tested genotypes, P2088 yielded the highest average grain yield (14.50 t ha^-1^), while KALUMET showed the latest flowering and the tallest plants. Protein content was the only compositional trait significantly impacted by genotype, with DKC6680 showing the highest average protein content (10.33%) and P2088 the lowest (9.16%). Conversely, mineral traits remained statistically stable across genotypes and years.

**Discussion:**

Overall, the findings provide preliminary information on the regional performance of six maize genotypes under Black Sea ecological conditions and may support further multi-location testing before cultivar recommendation.

## 1 Introduction

Maize (*Zea mays* L.) is among the world’s most valuable cereals, known for its high yields and versatility (Erenstein et al., 2022). It is essential in agriculture for various uses, including consumption, feed, starch, and biofuels (OECD/FAO, 2024). As the global population grows, demand for feed and raw materials increases, underscoring the need to develop superior genotypes with higher yields and quality (FAO, 2024). Testing new maize genotypes across different environments is vital for ensuring stable production and encouraging sustainable farming practices (Tester and Langridge, 2010). The species’ adaptability to various ecological conditions is linked to its wide genetic diversity and long domestication history (Matsuoka et al., 2002). Despite domestication and recent breeding efforts that have shaped maize’s genetics, significant variation remains in yield, morphology, and grain quality (Hufford et al., 2012). This genetic diversity is a valuable resource for selecting high-performing genotypes suited to diverse regions and for developing locally adapted varieties (Duvick, 2005). However, a genotype’s genetic potential may not always translate directly into field performance, as environmental factors significantly influence outcomes (Lobell et al., 2011). Environmental conditions, such as temperature, rainfall, soil structure, and climate fluctuations during the growing season, directly affect traits such as flowering time, plant development, kernel filling, and moisture content in maize (Yan et al., 2007). Genotype × environment interaction is a key source of variation in maize adaptation studies (Ndhlela et al., 2014). Multi-year and regional trials show genotype yield levels and responses to environmental changes (Yan and Tinker, 2006). Türkiye requires local testing of maize genotypes due to regional climate and soil differences (Şensoy et al., 2008). Maize’s significance for feed, grain, and industry underscores the need for high-yielding, high-quality genotypes (Tasdan, 2024), particularly in the Black Sea Region, where a humid climate, variable rainfall, and unique soils create a distinctive maize-growing environment with diverse genotype responses (Özata, 2020). Examining the agricultural performance and grain quality of new maize genotypes in Black Sea conditions is vital to find suitable materials. While grain yield is essential in maize selection, evaluations solely based on yield are now insufficient (Mahama et al., 2025). Protein, starch, fat, cellulose, ash, and minerals determine grain quality, directly affecting maize’s use in feed, food, and industry (Çiftçi et al., 2025). Protein and starch accumulation are influenced by genotypic structure, carbon and nitrogen metabolism, grain filling, and source-pool relationships (Seebauer et al., 2010). While grain oil content indicates energy value, ash and mineral content complement the grain’s nutritional profile (Mahama et al., 2025). Minerals such as Fe, Cu, Mn, Ca, and Mg provide greater insight into the nutritional quality and functional potential of maize kernels (Çiftçi et al., 2025). Evaluating agricultural performance and grain quality together offers a stronger basis for selecting suitable genotypes. Non-destructive techniques such as near-infrared reflectance spectroscopy enable quick, reliable quality checks, especially for large genotype sets (Jiang et al., 2007). Portable NIR devices and multivariate calibration facilitate high-capacity sampling for cereal chemical analysis (Simeone et al., 2024). Correlation analysis helps interpret relationships between maize genotype traits across agricultural and quality characteristics (Wei and Simko, 2024). Principal component analysis and biplot provide a multivariate performance visualization, supporting exploratory visualization of performance tendencies (Yan and Tinker, 2006). Assessing new maize genotypes in Türkiye will provide essential insights for regional genotype selection, evaluation, and further testing. This study aims to evaluate newly introduced maize genotypes within the Black Sea ecological zone, focusing on agricultural performance, grain quality, and mineral content, thus supporting the identification of promising materials for further regional performance testing.

## 2 Materials and methods

### 2.1 Experimental site and environmental conditions

The field experiment was conducted in Samsun-Bafra, Türkiye (41.60° N, 35.92° E), during the 2020 and 2021 growing seasons. Agrometeorological data, such as monthly average temperatures and total rainfall during the 2020 and 2021 growing seasons, were collected using the nasapower package in R (Sparks, 2018). This package fetches weather information from NASA POWER based on the geographic coordinates of the experimental site and the closest NASA POWER grid point. Monthly mean temperature and total precipitation were used to characterize environmental conditions during the study period. In both years, temperature followed a typical seasonal pattern, rising from spring to midsummer and then declining. Mean monthly temperature reached its highest values in July in both 2020 and 2021, indicating broadly similar thermal conditions during the major vegetative and reproductive stages. In contrast, precipitation distribution differed more markedly between years. Rainfall in 2020 was relatively concentrated in early summer, whereas in 2021 it was more uneven, with a pronounced peak in August. These differences indicate that, although thermal regimes were broadly comparable between years, the crop was exposed to contrasting moisture conditions during the growing season.

According to the Köppen-Geiger climate classification, the study area belongs to the Cfa climate type, characterized by mild winters, hot summers, and year-round precipitation (Şensoy et al., 2008). Based on the International Union of Soil Sciences classification system (IUSS, 2015), the experimental soil was classified as clay loam (CL), with approximately 45.3% loam, 29.2% clay, and 25.5% sand. The soil had a slightly alkaline reaction and a moderately calcareous structure. In addition, available phosphorus was insufficient, whereas organic matter content was low.

### 2.2 Experimental design, genotypes, and crop management

The experiment was established as a randomized complete block design (RCBD) with four replications in each year. Six maize genotypes were evaluated: HAV774, HAV911, HAV923, KALUMET, P2088, and DKC6680. Detailed information on genotype type, source/company or institute, country of origin, maturity group/FAO group, and introduction status is provided in **Supplementary Table 1**. HAV774, HAV911, and HAV923 were introduced dent-type genotypes, whereas KALUMET, P2088, and DKC6680 were registered dent-type genotypes included as check materials for benchmarking under the same field conditions.

Each experimental plot consisted of four rows, each 7 m long, with 70 cm inter-row spacing and 15 cm within-row plant spacing. Accordingly, the gross plot area was 19.6 m². At harvest, the two central rows were used for data collection and yield determination, corresponding to a net harvested plot area of 9.8 m².

Before sowing, diammonium phosphate (DAP; 18:46:0) fertilizer was applied at a rate of 100 kg ha^-1^. After sowing, 20:20:0 fertilizer was applied at 100 kg ha^-1^. Drip irrigation was applied at 5-day intervals throughout the growing period to maintain relatively stable soil moisture conditions. Each irrigation event continued until the soil approached field capacity, corresponding to approximately 50–60 mm of water per application.

Weed control was initiated by manual removal during the early growth stages and was followed by hoeing at the 3–5 leaf stage as weed pressure increased. Harvest was performed between 20 and 27 October, with minor adjustments made based on genotype maturity and prevailing weather conditions.

### 2.3 Trait measurements

The evaluated variables included agronomic, morphological, compositional, and mineral traits. Agronomic and morphological traits comprised days to pollen shedding (Pollen DAP), plant height, ear height, grain moisture content (%), and grain yield (t ha^-1^). Compositional traits included ash (%), fat (%), protein (%), cellulose (%), and starch (%), whereas mineral traits included concentrations of Fe, Cu, Mn, Ca, and Mg.

The principal field observations, including days to pollen shedding, plant height, ear height, grain moisture content, and yield, were recorded according to the standard operating procedures published by the Seed Certification Center of Türkiye (TTSM, 2025). Pollen DAP was defined as the number of days from sowing to 50% pollen shedding within a plot. Plant height was measured from the soil surface to the tassel tip, whereas ear height was measured from the soil surface to the node bearing the uppermost ear. Grain moisture content and grain yield were determined at harvest from the net harvested area.

Nutrient composition traits, namely cellulose (%), starch (%), fat (%), ash (%), and protein (%), were predicted by near-infrared reflectance spectroscopy near-infrared reflectance spectroscopy (NIRS) using a FOSS NIR Systems 6500 scanning monochromator spectrometer (FOSS A/S, Hillerød, Denmark). Spectral data were collected over the 1100–2498 nm wavelength range at 2-nm intervals, and constituent predictions were obtained using the IC-0904FE calibration/software package.

Phosphorus (P), potassium (K), calcium (Ca), magnesium (Mg), iron (Fe), copper (Cu), zinc (Zn), and manganese (Mn) levels were measured after dry ashing. Samples were ashed at 500–550 °C in a furnace until gray ash without carbonized particles. The ash was dissolved in 4 mL of 3 N HNO3, and the extracts analyzed by Inductively Coupled Plasma Optical Emission Spectrometry (ICP-OES) (Huang and Schulte, 1985; Jones, 2001; Khan et al., 2022).

For ICP-OES analysis, calibration was performed using element-specific standard solutions before sample measurement. Blank samples were included throughout the analytical procedure to monitor background signal and possible contamination, thereby supporting the reliability of mineral concentration measurements.

### 2.4 Statistical analysis of plant traits

Trait data were analyzed using a linear mixed-effects model. In the combined analysis, genotype was treated as a fixed effect, whereas year, replication within year, and the genotype × year interaction were considered random effects. The model was expressed as:


$$y_{ij}=\mu +G_{i}+Y_{j}+{\left(G\times Y\right)}_{ij}+\epsilon_{ij}$$

where $y_{ij}$ is the observed value of the trait, μ is the overall mean, $G_{i}$ is the fixed effect of the i-th genotype treatment, $Y_{j}$ is the random effect of the j-th year, ${\left(G\times Y\right)}_{ij}$ is the random interaction effect between genotype and year, and $\epsilon_{ij}$ is the residual error.

Genotype was a fixed effect to compare tested materials, while year and replication accounted for environmental and experimental variation across seasons.

Year-specific and combined means across years were estimated from the fitted models. When the genotype effect was significant at P < 0.05, pairwise comparisons among means were performed using Tukey’s multiple comparison test, and compact letter displays were used to separate means. For traits with a non-significant genotype × year interaction, genotype groupings were interpreted across years, whereas traits with significant interaction were interpreted within year. Significance levels were reported as ns (not significant), * for P < 0.05, ** for P < 0.01, and *** for P < 0.001.

The coefficient of variation (CV, coefficient of variation) was calculated as:


$$Cv(\%)=\frac{\sigma_{e}}{\bar{x}}\times 100$$

where $\sigma_{e}$​ is the residual standard deviation and $\bar{x}$ is the overall trait mean.

### 2.5 Correlation analysis and principal component analysis of plant traits

Correlation analysis and PCA served as exploratory tools to visualize relationships among traits and summarize performance across genotype-year combinations, given the limited number of genotypes. Pearson correlation coefficients provided a descriptive overview of trait associations across the dataset and within each year, visualized as heatmaps. PCA on standardized means reduced the influence of differing measurement scales and facilitated the interpretation of the distributions of genotype-year combinations and trait vectors. PCA results were interpreted alongside and subordinate to mixed-model analysis and genotype mean comparisons.

### 2.6 Software and data presentation

All statistical analyses were conducted in R (R Core Team, 2026). Linear mixed-effects models were fitted using the lme4 package (Bates et al., 2015), and fixed-effect significance testing was performed using lmerTest (Kuznetsova et al., 2017). Estimated marginal means and Tukey-adjusted pairwise comparisons were obtained using emmeans (Lenth and Piaskowski, 2026). Data import and preprocessing were carried out using readxl (Wickham and Bryan, 2025) and dplyr (Wickham et al., 2026). Figures were prepared primarily with ggplot2 (Wickham, 2016), whereas multivariate visualizations were generated using factoextra (Kassambara and Mundt, 2026). Correlation heatmaps were produced using corrplot (Wei and Simko, 2024).

## 3 Results

**Figure 1:**
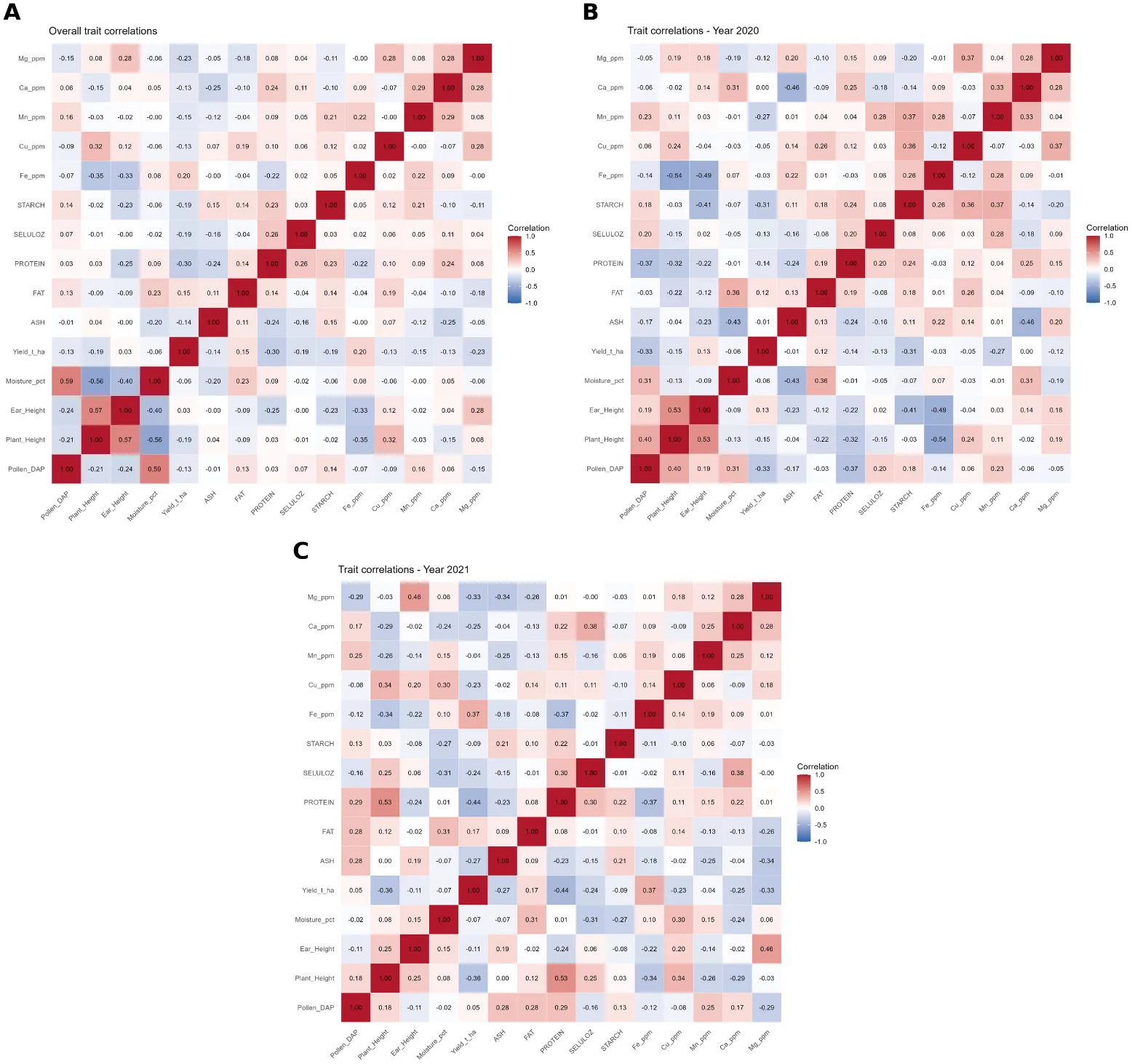
Exploratory correlation heatmaps depict relationships among phenological, agronomic, quality, and mineral traits in maize. **(A)** shows the overall correlation matrix; **(B, C)** show matrices for 2020 and 2021. Correlation coefficients are displayed within cells, with red for positive and blue for negative correlations.

**Figure 2:**
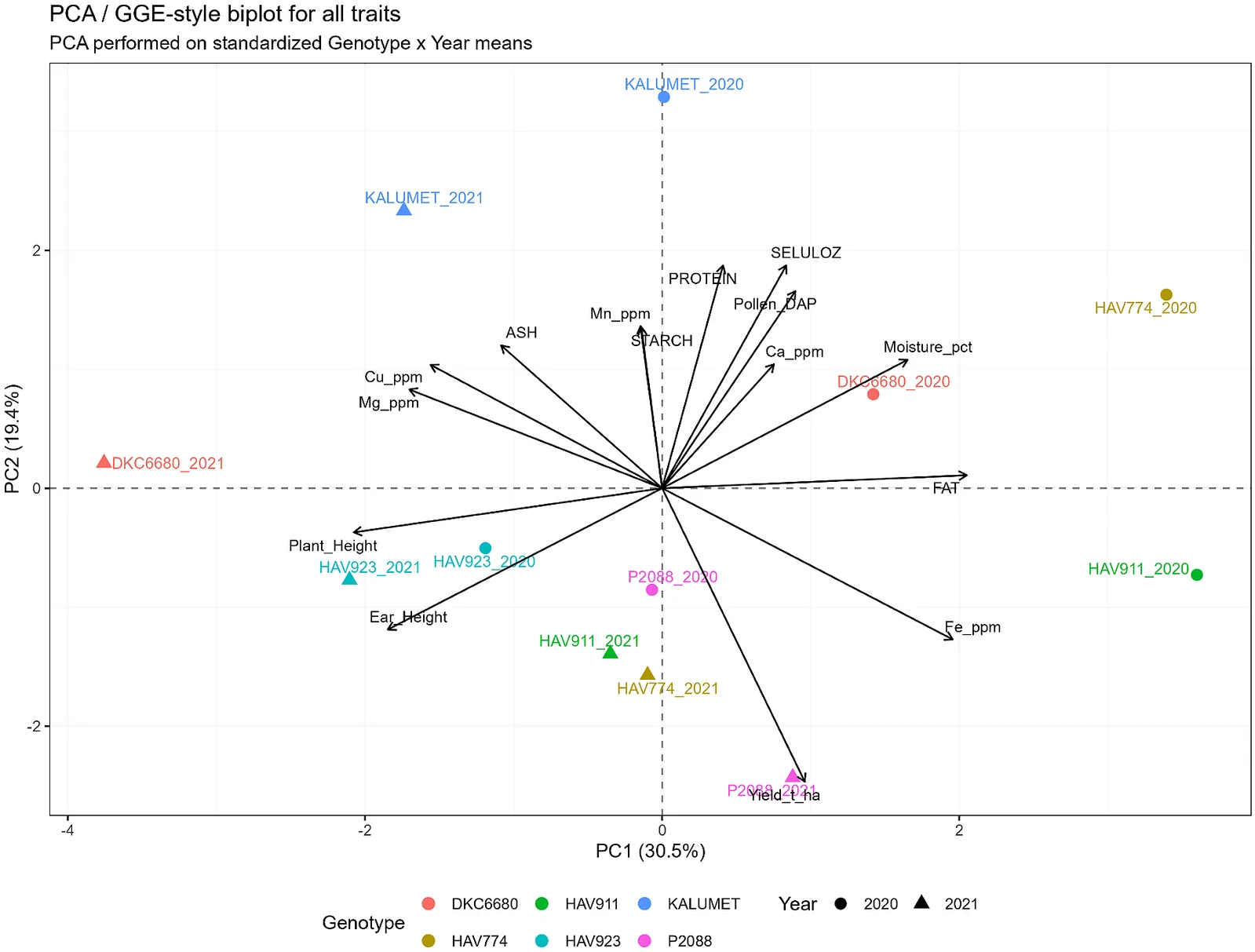
Exploratory PCA biplot shows the distribution of genotype-year combinations and traits based on standardized means. The first two components explain 30.5% and 19.4% of the variation. Genotypes are color-coded; years are symbols. Trait vectors indicate associations with principal components.

**Figure 3:**
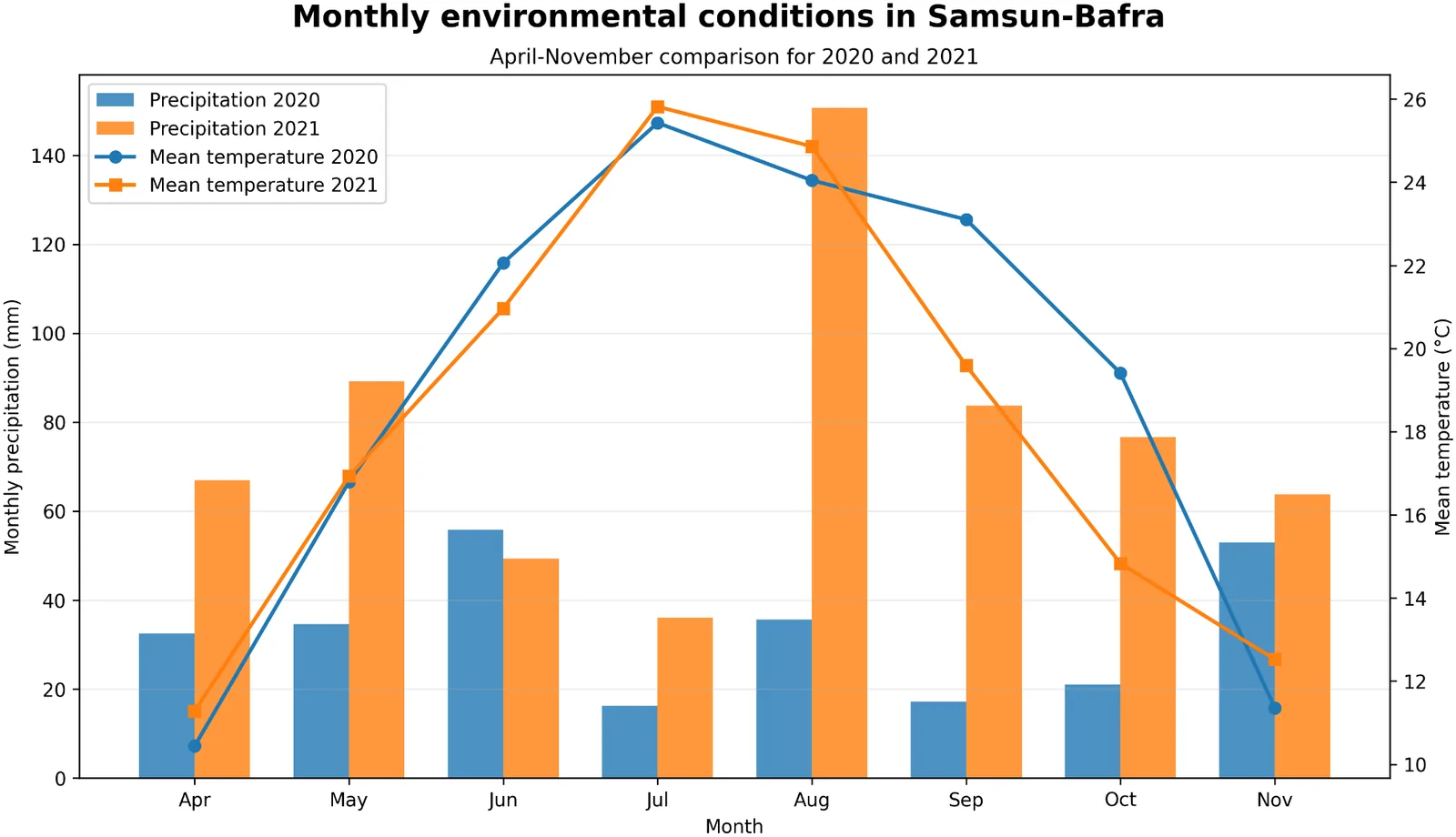
Monthly mean temperature and total precipitation recorded during the 2020 and 2021 growing seasons in Samsun-Bafra, Türkiye. Bars indicate monthly total precipitation, and the line represents mean monthly temperature.

**Figure 4:**
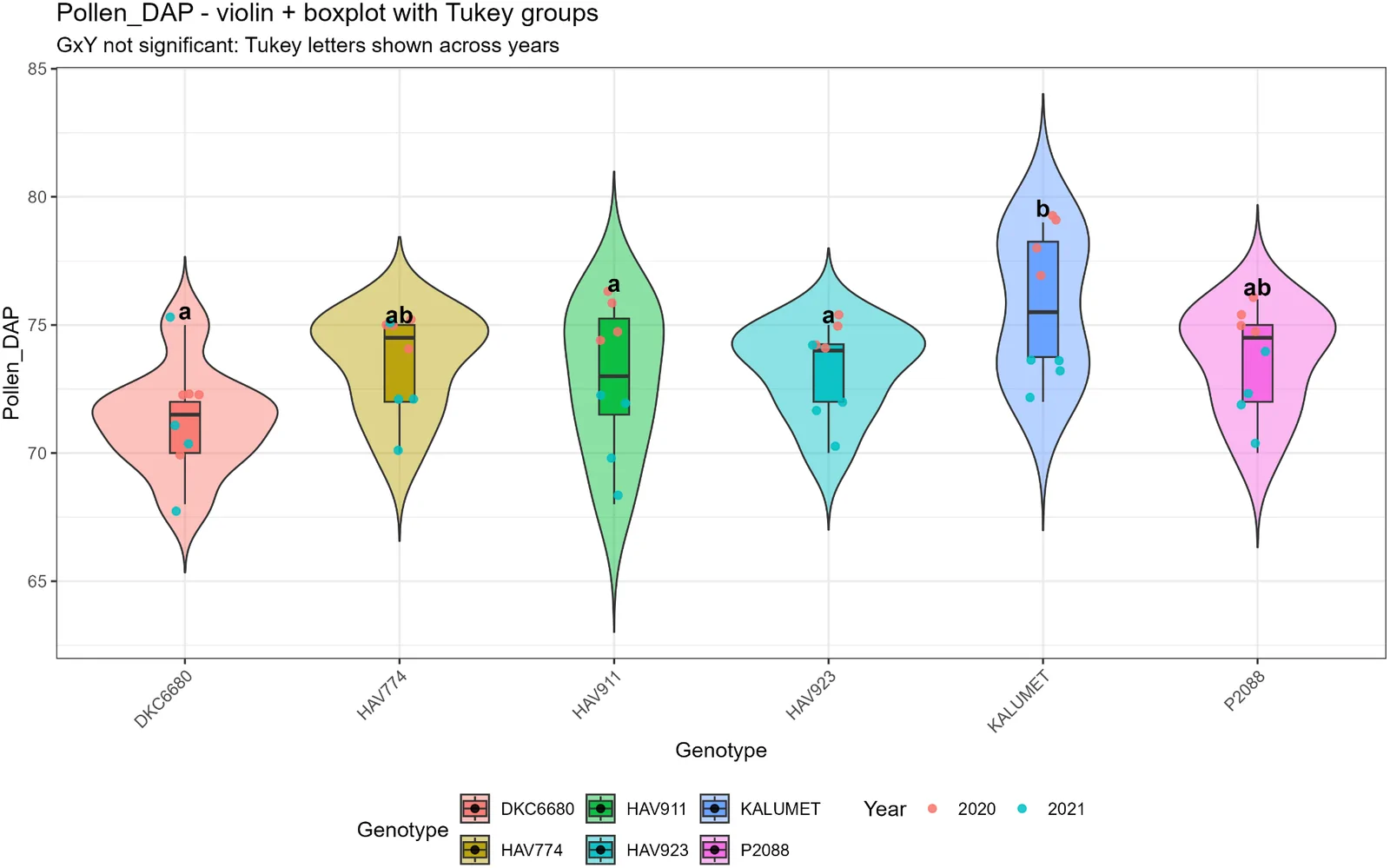
Distribution of days to pollen shedding (Pollen_DAP) among maize genotypes based on combined data from 2020 and 2021. Violin plots represent the trait distribution, boxplots show median and dispersion, and points correspond to year-specific observations. As the genotype × year interaction was not significant, Tukey grouping letters are given across years; genotypes sharing at least one letter are not significantly different at P < 0.05.

**Figure 5:**
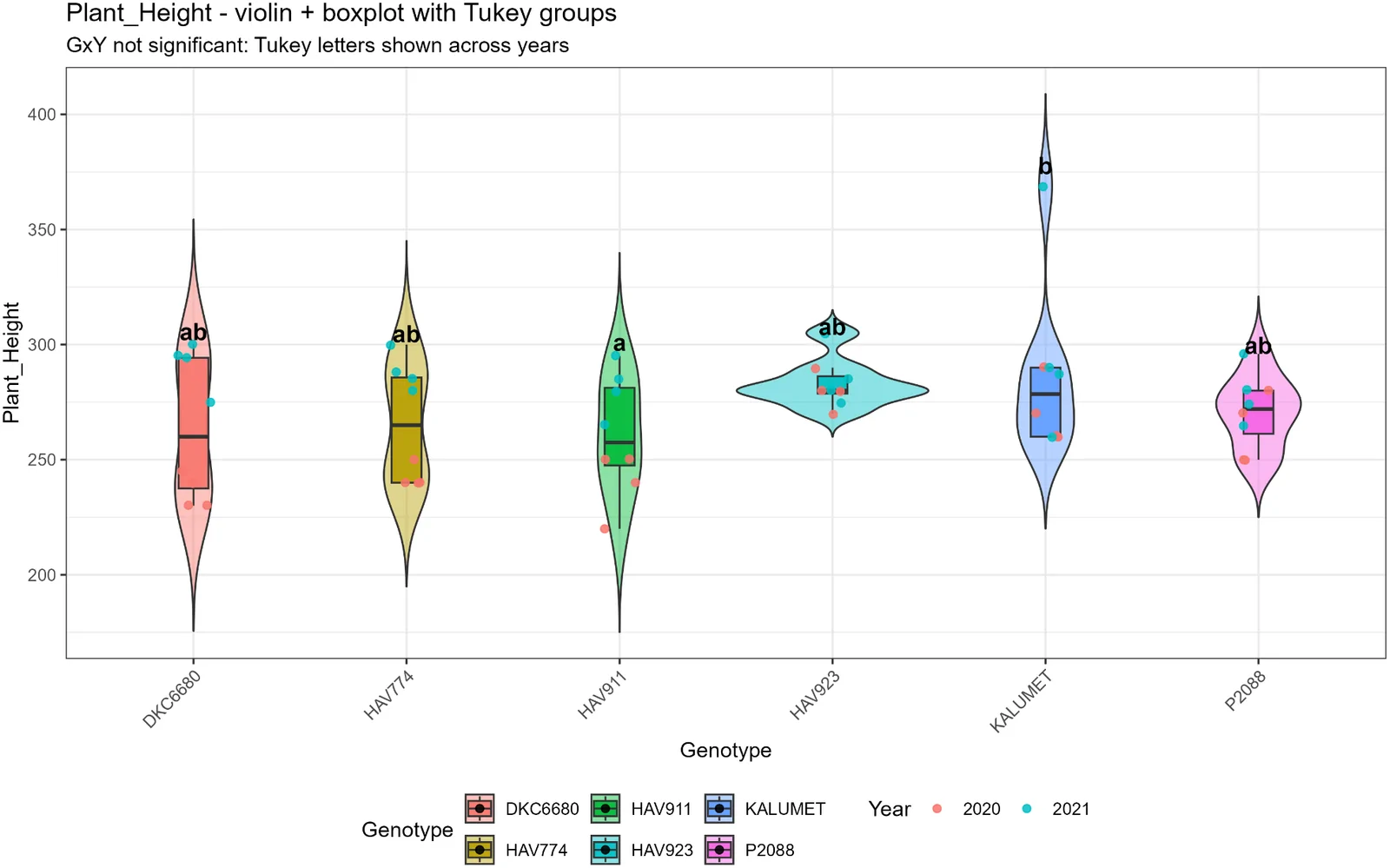
Distribution of plant height among maize genotypes based on combined data from 2020 and 2021. Violin plots illustrate the distribution pattern, boxplots summarize the central tendency and variability, and points indicate individual yearly values. Because the genotype × year interaction was not significant, Tukey grouping letters are shown across years; genotypes with at least one common letter do not differ significantly at P < 0.05.

**Figure 6:**
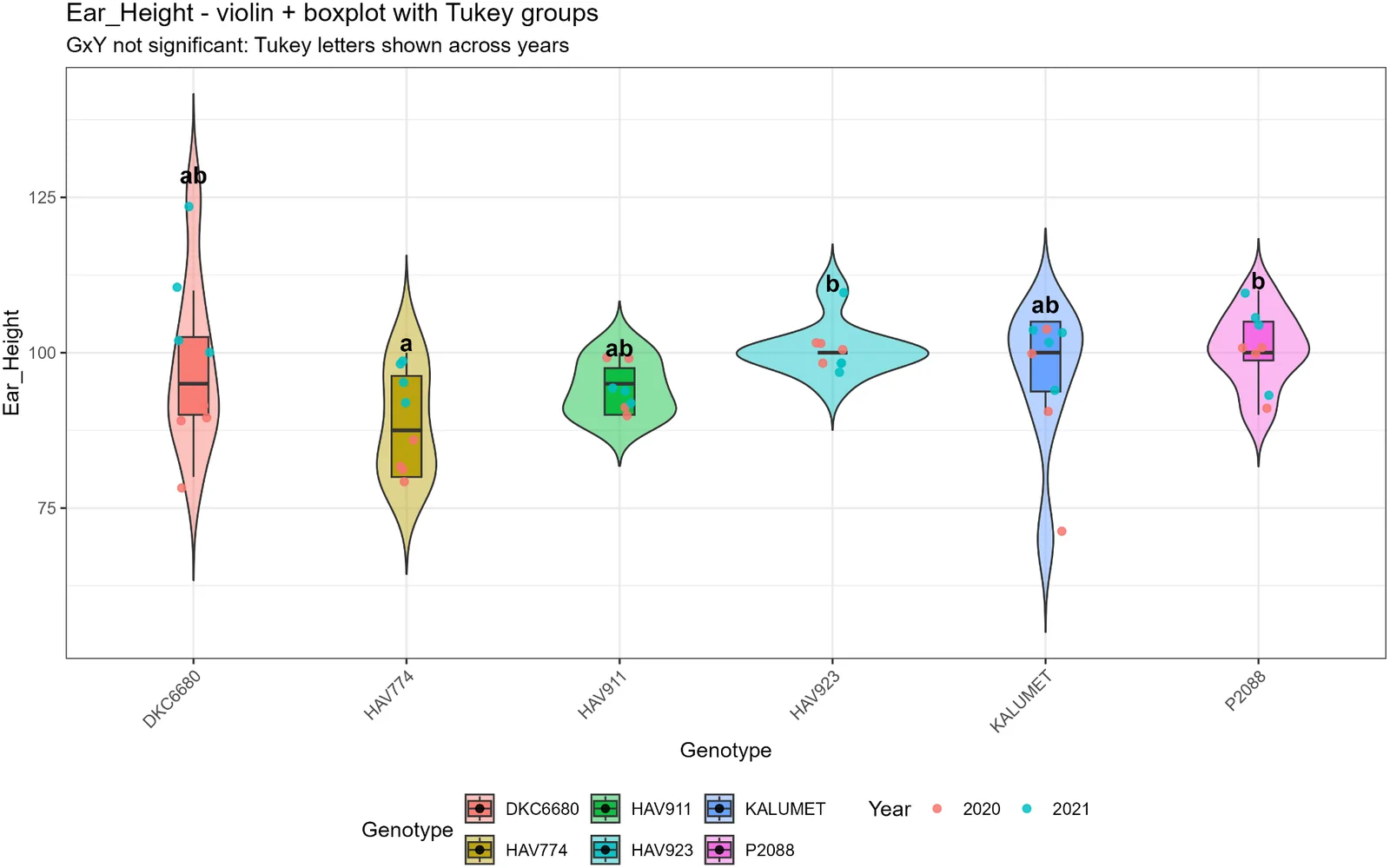
Distribution of ear height among maize genotypes based on combined data from 2020 and 2021. Violin plots show the density distribution, boxplots indicate the median and interquartile range, and points represent individual yearly observations. Since the genotype × year interaction was not significant, Tukey grouping letters are presented across years; genotypes sharing at least one letter are not significantly different at P < 0.05.

**Figure 7:**
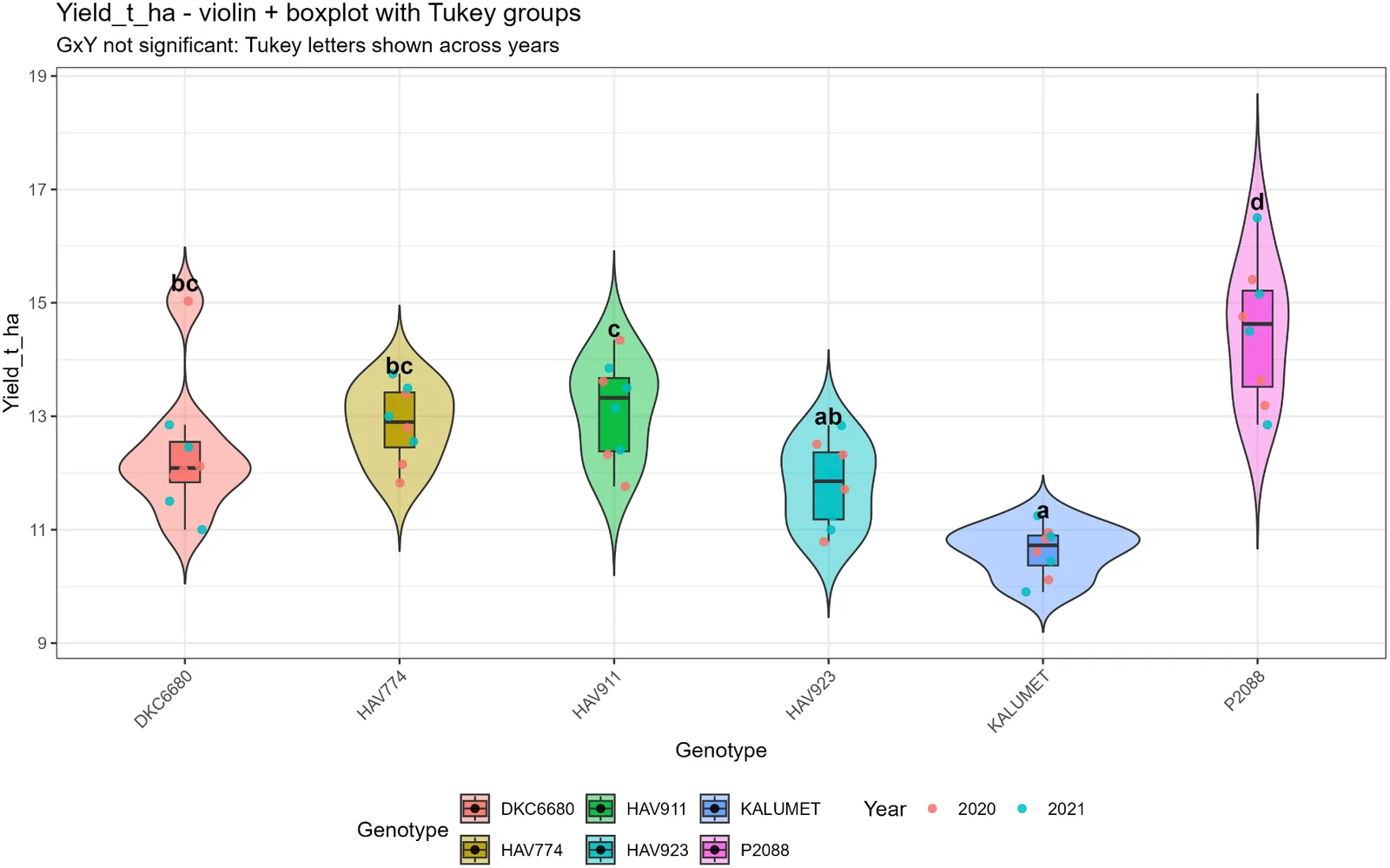
Distribution of grain yield (t ha^-1^) among maize genotypes based on combined data from 2020 and 2021. Violin plots indicate the distribution of values, boxplots summarize the median and spread, and points represent individual yearly observations. Because the genotype × year interaction was not significant, Tukey grouping letters are shown across years; genotypes sharing at least one letter are not significantly different at *P* < 0.05.

The exploratory correlation heatmaps, PCA biplot, and monthly environmental conditions are presented in **Figures 1**–**3**, respectively. Significant genotype and year effects were detected for several agronomic traits (**Table 1**). Days to pollen shedding (Pollen DAP) differed among genotypes and between years, with the overall mean being higher in 2020 than in 2021 (74.9 vs. 71.8 days, *P* < 0.0001). KALUMET had the highest mean Pollen DAP (75.8 days), while HAV911, HAV923, and DKC6680 were among the earliest genotypes. Plant height and ear height were also significantly affected by genotype and year, with taller plants and higher ear placement generally observed in 2021 than in 2020. KALUMET had the highest mean plant height (285.8 cm), whereas HAV911 had the lowest (260.6 cm). For ear height, HAV923 and P2088 had the highest means (100.6 cm), while HAV774 had the lowest mean ear height (88.8 cm). Grain moisture was significantly affected by year and by the genotype × year interaction, declining from 24.38% in 2020 to 19.94% in 2021. In contrast, grain yield was significantly influenced by genotype but not by year or genotype × year interaction; P2088 produced the highest mean yield (14.50 t ha^-1^), followed by HAV911 (13.12 t ha^-1^), whereas KALUMET had the lowest mean yield (10.62 t ha^-1^) (**Table 1**; **Figures 4**–**7**).

**Table 1:** Agronomic traits of maize genotypes evaluated in 2020 and 2021.

Genotype	Pollen DAP			Plant height (cm)			Ear height (cm)			Moisture (%)			Yield (t ha^-1^)		
	2020	2021	Mean	2020	2021	Mean	2020	2021	Mean	2020	2021	Mean	2020	2021	Mean
HAV774	74.8 ± 0.2	72.2 ± 1.0	73.5 ± 0.7 ab	242.5 ± 2.5	288.2 ± 4.2	265.4 ± 8.9 ab	81.2 ± 1.2	96.2 ± 2.4	88.8 ± 3.1 a	26.12 ± 0.28	19.43 ± 0.58	22.77 ± 1.30	12.54 ± 0.35	13.20 ± 0.27	12.87 ± 0.24 bc
HAV911	75.2 ± 0.5	70.5 ± 1.0	72.9 ± 1.0 a	240.0 ± 7.1	281.2 ± 6.2	260.6 ± 8.9 a	95.0 ± 2.9	93.3 ± 1.7	94.3 ± 1.7 ab	25.80 ± 0.31	20.00 ± 0.18	22.90 ± 1.11	13.01 ± 0.59	13.22 ± 0.31	13.12 ± 0.31 c
HAV923	74.5 ± 0.3	72.0 ± 0.8	73.2 ± 0.6 a	280.0 ± 4.1	286.2 ± 6.6	283.1 ± 3.8 ab	100.0 ± 0.0	101.2 ± 3.1	100.6 ± 1.5 b	24.43 ± 0.39	20.50 ± 0.67	22.46 ± 0.82	11.83 ± 0.39	11.77 ± 0.42	11.80 ± 0.26 ab
KALUMET	78.2 ± 0.5	73.2 ± 0.5	75.8 ± 1.0 b	270.0 ± 7.1	301.5 ± 23.5	285.8 ± 12.8 b	91.2 ± 7.7	101.2 ± 2.4	96.2 ± 4.2 ab	24.12 ± 0.31	19.80 ± 0.86	21.96 ± 0.92	10.63 ± 0.18	10.62 ± 0.29	10.62 ± 0.16 a
P2088	75.2 ± 0.2	72.0 ± 0.8	73.6 ± 0.7 ab	262.5 ± 7.5	278.8 ± 6.5	270.6 ± 5.5 ab	97.5 ± 2.5	103.8 ± 3.1	100.6 ± 2.2 b	23.27 ± 0.17	19.98 ± 0.48	21.62 ± 0.67	14.25 ± 0.51	14.75 ± 0.76	14.50 ± 0.43 d
DKC6680	71.5 ± 0.5	71.0 ± 1.5	71.2 ± 0.7 a	236.2 ± 3.8	291.0 ± 5.5	263.6 ± 10.8 ab	87.5 ± 2.5	108.8 ± 5.9	98.1 ± 5.0 ab	22.52 ± 0.41	19.93 ± 1.14	21.23 ± 0.74	12.79 ± 0.75	11.95 ± 0.42	12.37 ± 0.43 bc
**Mean**	74.9 A	71.8 B		255.2 B	287.8 A		92.1 B	101.1 A		24.38 A	19.94 B		12.51	12.59	
**G**	<0.0001*			0.0111*			0.0297*			0.3513			<0.0001*		
**Y**	<0.0001*			<0.0001*			0.0002*			<0.0001*			0.7581		
**G × Y**	0.1246			0.0561			0.0870			0.0154*			1.0000		
**CV (%)**	2.21			5.43			7.46			4.82			6.91		

An asterisk (*) indicates a statistically significant effect at P < 0.05. Values are presented as mean ± SE. Lowercase letters in the Mean column indicate Tukey HSD genotype groups for traits with significant genotype effects and non-significant G × Y interaction. Uppercase letters in the bottom Mean row indicate significant year differences (P < 0.05). G, genotype effect; Y, year effect; G × Y, genotype × year interaction; CV, coefficient of variation. Bold P-values indicate statistically significant effects (P < 0.05).

The violin-box plots supported the ANOVA results and illustrated the dispersion of genotype performance across years (**Figures 4**–**7**). Because genotype × year interaction was not significant for Pollen DAP, plant height, ear height, and yield, Tukey groupings were interpreted across years. KALUMET had the highest Pollen DAP group, whereas P2088 had the highest yield group. For plant height, KALUMET was in the highest group, while HAV911 was in the lower groups. Ear height showed a similar pattern, with HAV923 and P2088 tending to occupy the upper groups and HAV774 the lower group. Overall, these figures indicate that genotype differences were more consistent than year-specific rank changes for these traits.

**Figure 8:**
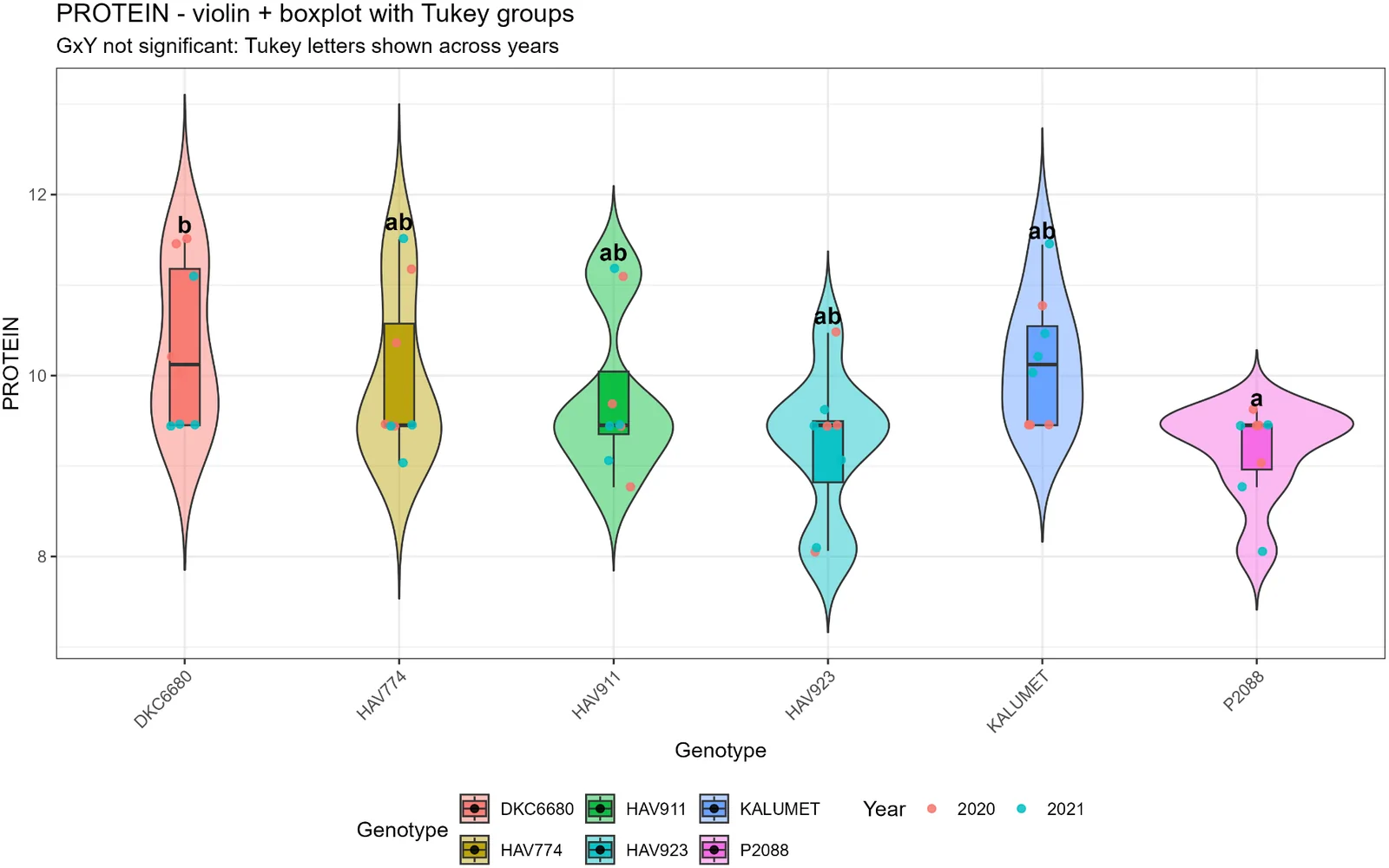
Distribution of protein content among maize genotypes based on combined data from 2020 and 2021. Violin plots display the distribution shape, boxplots summarize variation, and points show the observations for each year. Since the genotype × year interaction was not significant, Tukey grouping letters are presented across years; means with at least one common letter are not significantly different at *P* < 0.05.

Among proximate composition traits, only protein content showed a significant genotype effect (**Table 2**). DKC6680 had the highest mean protein content (10.33%), whereas P2088 had the lowest (9.16%). Ash, fat, cellulose, and starch were not significantly affected by genotype, year, or genotype × year interaction, indicating relative stability of these traits across the two growing seasons. The distribution of protein values shown in **Figure 8** was consistent with the mean comparisons in **Table 2**, with DKC6680 tending toward higher values and P2088 toward lower values.

**Table 2:** Proximate composition traits of maize genotypes evaluated in 2020 and 2021.

Genotype	Ash (%)			Fat (%)			Protein (%)			Cellulose (%)			Starch (%)		
	2020	2021	Mean	2020	2021	Mean	2020	2021	Mean	2020	2021	Mean	2020	2021	Mean
HAV774	2.13 ± 0.01	2.08 ± 0.04	2.10 ± 0.02	4.50 ± 0.27	4.12 ± 0.12	4.31 ± 0.16	10.11 ± 0.42	9.86 ± 0.56	9.98 ± 0.33 ab	3.20 ± 0.43	3.07 ± 0.46	3.13 ± 0.29	70.92 ± 0.70	68.89 ± 1.94	69.91 ± 1.03
HAV911	1.97 ± 0.09	2.12 ± 0.00	2.05 ± 0.05	4.42 ± 0.42	4.25 ± 0.14	4.34 ± 0.21	9.75 ± 0.49	9.78 ± 0.47	9.77 ± 0.31 ab	3.26 ± 0.32	2.81 ± 0.35	3.04 ± 0.24	68.49 ± 0.73	71.00 ± 0.87	69.75 ± 0.71
HAV923	2.12 ± 0.00	2.12 ± 0.00	2.12 ± 0.00	3.94 ± 0.15	4.18 ± 0.08	4.06 ± 0.09	9.36 ± 0.49	9.06 ± 0.34	9.21 ± 0.28 ab	2.91 ± 0.46	2.70 ± 0.26	2.80 ± 0.25	68.78 ± 1.13	69.56 ± 1.29	69.17 ± 0.81
KALUMET	2.12 ± 0.00	2.23 ± 0.08	2.17 ± 0.04	4.21 ± 0.06	4.15 ± 0.10	4.18 ± 0.06	9.78 ± 0.33	10.54 ± 0.32	10.16 ± 0.26 ab	3.45 ± 0.35	3.50 ± 0.50	3.47 ± 0.28	71.23 ± 0.92	68.60 ± 0.54	69.92 ± 0.70
P2088	2.16 ± 0.04	2.12 ± 0.01	2.14 ± 0.02	4.12 ± 0.05	4.36 ± 0.46	4.24 ± 0.22	9.39 ± 0.13	8.93 ± 0.33	9.16 ± 0.19 a	2.96 ± 0.39	3.28 ± 0.46	3.12 ± 0.29	69.16 ± 2.02	68.59 ± 1.07	68.88 ± 1.07
DKC6680	2.16 ± 0.11	2.12 ± 0.12	2.14 ± 0.08	4.23 ± 0.03	4.00 ± 0.00	4.12 ± 0.05	10.80 ± 0.39	9.86 ± 0.41	10.33 ± 0.32 b	3.39 ± 0.47	3.09 ± 0.36	3.24 ± 0.28	69.38 ± 0.33	70.42 ± 1.18	69.90 ± 0.60
**Mean**	2.11	2.13		4.24	4.18		9.86	9.67		3.19	3.07		69.66	69.51	
**G**	0.3706			0.6855			0.0093*			0.5553			0.8898		
**Y**	0.6001			0.6504			0.3887			0.5960			0.8170		
**G × Y**	1.0000			1.0000			0.9828			1.0000			1.0000		
**CV (%)**	5.95			10.13			7.80			24.83			3.19		

An asterisk (*) indicates a statistically significant effect at P < 0.05. Values are presented as mean ± SE. Lowercase letters in the Mean column indicate Tukey HSD genotype groups for traits with significant genotype effects and non-significant G × Y interaction. Uppercase letters in the bottom Mean row indicate significant year differences (P < 0.05). G, genotype effect; Y, year effect; G × Y, genotype × year interaction; CV, coefficient of variation. Bold P-values indicate statistically significant effects (P < 0.05).

Mineral traits were comparatively stable across genotypes and years (**Table 3**). Fe, Cu, Mn, Ca, and Mg concentrations were not significantly affected by genotype, year, or genotype × year interaction. Although numerical differences were observed among genotypes, especially for Ca and Mg, they were not statistically significant, suggesting that mineral composition was less responsive than agronomic performance under the conditions of this study.

**Table 3:** Mineral traits of maize genotypes evaluated in 2020 and 2021.

Genotype	Fe (ppm)			Cu (ppm)			Mn (ppm)			Ca (ppm)			Mg (ppm)		
	2020	2021	Mean	2020	2021	Mean	2020	2021	Mean	2020	2021	Mean	2020	2021	Mean
HAV774	24.50 ± 1.74	22.69 ± 3.25	23.60 ± 1.74	6.04 ± 0.59	5.36 ± 0.37	5.70 ± 0.35	6.24 ± 0.60	5.40 ± 0.89	5.82 ± 0.52	473.89 ± 198.94	373.96 ± 219.19	423.93 ± 138.32	1037.20 ± 29.38	1102.05 ± 37.95	1069.62 ± 25.37
HAV911	23.57 ± 2.64	22.66 ± 3.93	23.11 ± 2.20	5.41 ± 0.87	6.64 ± 0.78	6.02 ± 0.59	5.06 ± 0.54	5.19 ± 0.36	5.13 ± 0.30	800.06 ± 690.63	104.88 ± 20.62	452.47 ± 345.77	1053.25 ± 116.36	1008.41 ± 48.83	1030.83 ± 59.02
HAV923	20.24 ± 1.72	21.78 ± 1.75	21.01 ± 1.17	5.95 ± 0.48	6.77 ± 0.78	6.36 ± 0.45	5.25 ± 0.72	6.26 ± 0.61	5.75 ± 0.48	235.92 ± 49.79	156.87 ± 45.68	196.40 ± 34.66	1113.54 ± 44.69	1148.14 ± 45.68	1130.84 ± 30.30
KALUMET	20.42 ± 1.78	20.50 ± 1.44	20.46 ± 1.06	6.59 ± 0.81	6.78 ± 0.28	6.69 ± 0.40	6.50 ± 0.58	5.79 ± 0.64	6.15 ± 0.42	265.99 ± 87.77	1059.75 ± 621.42	662.87 ± 326.96	1121.29 ± 40.70	1091.76 ± 79.83	1106.52 ± 41.85
P2088	22.27 ± 2.07	25.34 ± 2.16	23.80 ± 1.50	6.43 ± 0.52	6.07 ± 0.59	6.25 ± 0.37	5.25 ± 0.63	6.22 ± 0.79	5.73 ± 0.50	332.17 ± 164.02	273.73 ± 112.25	302.95 ± 92.67	1116.38 ± 53.95	1001.89 ± 12.46	1059.14 ± 33.54
DKC6680	22.38 ± 2.20	17.89 ± 1.62	20.14 ± 1.52	5.73 ± 0.54	6.57 ± 0.57	6.15 ± 0.40	5.47 ± 1.04	5.64 ± 1.03	5.55 ± 0.68	219.73 ± 119.56	116.37 ± 15.84	168.05 ± 59.15	1106.81 ± 117.15	1234.93 ± 87.24	1170.87 ± 71.82
**Mean**	22.23	21.81		6.03	6.37		5.63	5.75		387.96	347.59		1091.41	1097.86	
**G**	0.2041			0.4102			0.7681			0.5036			0.2628		
**Y**	0.7218			0.2230			0.7762			0.8113			0.8709		
**G × Y**	1.0000			0.8998			1.0000			1.0000			1.0000		
**CV (%)**	18.34			15.26			26.12			157.85			12.46		

Values are presented as mean ± SE. Lowercase letters in the Mean column indicate Tukey HSD genotype groups for traits with significant genotype effects and non-significant G × Y interaction. Uppercase letters in the bottom Mean row indicate significant year differences (P < 0.05). G, genotype effect; Y, year effect; G × Y, genotype × year interaction; CV, coefficient of variation. Bold P-values indicate statistically significant effects (P < 0.05).

Correlation analysis summarized the relationships among agronomic, compositional, and mineral traits (**Figure 1**). Pollen DAP was generally positively associated with grain moisture, while plant height correlated with ear height. Grain yield tended to negatively relate to protein content. Due to the limited number of genotypes, these findings are cautious and not causal. Year-specific heatmaps showed how associations varied across seasons.

The PCA biplot summarized genotype-year distributions across traits. The first two components explained 49.9% of the variation, with PC1 accounting for 30.5% and PC2 for 19.4%. P2088_2021 was positioned near the grain yield vector, which was consistent with its high yield in the mixed-model mean comparison. KALUMET entries were more closely associated with delayed flowering and plant height traits, whereas DKC6680_2021 and HAV923_2021 were more closely associated with vegetative growth traits. These patterns were exploratory, with primary interpretations based on mixed-model results and the means in **Tables 1**-**3**.

## 4 Discussion

The variations in flowering time, plant and ear height, and grain yield among new maize genotypes indicate that agronomic performance is influenced by genotype (Bocianowski et al., 2024). The genotype effect on agronomic traits shows performance differences among tested materials under Black Sea conditions, but these should be seen as regional testing results rather than indicating broad genetic variability (Magar et al., 2021).

Because maize grain yield is complex, evaluating genotypes by yield, phenology, plant architecture, and grain quality offers a more accurate approach (Long et al., 2024).

The differences in flowering time among genotypes suggest newly introduced materials should be carefully evaluated for suitability to maturity groups and regional calendars (Katsenios et al., 2021). Late-flowering genotypes may have longer vegetative periods but this does not always lead to higher grain yield (Long et al., 2024). The study also shows that variation in flowering time across years indicates that phenological development is sensitive to temperature, rainfall, and environmental conditions (Imran et al., 2024).

Genotype and year notably influence plant and ear height, reflecting genetic and environmental controls on maize growth (Wen et al., 2025). A positive link between plant and ear height suggests that maize architecture is a developmental trait, with taller plants often having higher ears (Nzuve et al., 2014). However, very high ear placement can cause lodging, reduce harvesting efficiency, and compromise stability. Thus, optimizing ear height is essential to balance yield and stability.

The notable interaction between year and genotype × year in grain moisture reveals that moisture content at harvest varies not only by genotype but also with environmental conditions that change yearly (Greveniotis et al., 2023). Harvest moisture level serves as a practical adaptation indicator along with yield when evaluating new genotypes, as it influences drying costs, storage safety, and optimal harvest timing in maize (Katsenios et al., 2021). In this research, the significant impact of genotype on grain yield, coupled with the non-significant effects of year and the genotype × year interaction, suggests that yield differences were mainly associated with the tested genotype effect under the present experimental conditions (Bocianowski et al., 2024). The minimal genotype × year interaction for yield indicates that some genotypes consistently performed similarly across both years, which is useful for preliminary regional performance assessment (Beyene et al., 2025).

In maize, high and stable grain yield is key for assessing the production potential of new genotypes across environments (Bocianowski et al., 2024). Some genotypes with high plant height did not top yield groups, indicating vegetative growth alone does not explain grain yield (Mazloom et al., 2025). Grain yield is determined not by individual traits such as plant or ear height, but rather by the combined effects of grain number, grain weight, ear structure, assimilation efficiency, and environmental adaptation (Long et al., 2024).

The genotypic effect noted only in protein content suggests that protein ratio is more sensitive to genotypic differences among quality components (Ertiro et al., 2022). In maize, protein, starch, and fat contents vary with genotype, environment, and conditions, so not all quality traits vary equally (Bojtor et al., 2022). Identifying high-protein genotypes is crucial for feed and nutritional quality, as grain protein influences maize’s use (Popović et al., 2025). The study shows high-yielding and high-protein genotypes differ, emphasizing the need to balance traits when selecting for yield and quality (Ertiro et al., 2022). The inverse relationship between yield and protein suggests that protein concentration may dilute with increasing grain dry matter (Greveniotis et al., 2023).

Genotype or year had no significant effect on ash, fat, cellulose, and starch, indicating these components are more stable than protein across environments (Katsenios et al., 2021). Grain quality resilience to environmental changes emphasizes the importance of evaluating new genotypes for consistent quality, not just high yield (Greveniotis et al., 2023). The lack of significant differences in Fe, Cu, Mn, Ca, and Mg suggests that mineral composition was relatively stable among the tested materials under the present experimental conditions (Bojtor et al., 2022). Mineral content varies with genotype, fertilization, soil nutrients, and environment; the absence of differences does not mean insignificance, as more variation may exist across a broader range of genotypes and soils (Dragicevic et al., 2016).

The analysis showed later-maturing genotypes have higher grain moisture at harvest (Greveniotis et al., 2023). The positive link between plant and ear height aligns with expected vegetative growth and ear placement (Wuhaib et al., 2018). A negative trend between yield and protein suggests a yield–quality trade-off, implying focusing only on yield might miss quality differences (Ertiro et al., 2022). Evaluating high-yield genotypes alongside grain quality traits can help make more balanced recommendations for producers and industry (Popović et al., 2025).

The exploratory PCA depicted the distribution of genotype-year combinations, providing a visual overview of performance trends that complemented the univariate results (**Figure 2**; Long et al., 2024). The varied directions of genotypes concerning yield, flowering, architecture, and quality highlighted descriptive performance differences among the tested materials, without indicating broad genetic separation or trait-specific patterns (Mazloom et al., 2025). When interpreted carefully, these analyses may provide visual support for materials showing high yield, suitable maturity, lower harvest moisture, and acceptable quality. However, the main conclusions should still be based on mixed-model results and mean comparisons (Long et al., 2024).

This study shows new maize genotypes in Türkiye vary in performance under Black Sea conditions, with some grain quality and mineral traits being less variable (Katsenios et al., 2021). Regional recommendations should consider yield, adaptability, harvest moisture, plant architecture, and grain quality (Beyene et al., 2025). Two-year studies help assess genotype responses, but more locations and years could better reveal their adaptive capacity (Bocianowski et al., 2024; Popović et al., 2025).

The study offers a regional performance evaluation of tested maize genotypes, not a broad genetic or trait-dissection study. The limited number of genotypes and specific Black Sea conditions mean the findings are applicable only to these materials and this environment. Broader conclusions about genetic diversity, trait stability, and adaptability require larger datasets and broader testing. However, the results provide useful preliminary data on agronomic traits such as yield, moisture, protein, and grain quality.

## 5 Conclusion

The evaluated maize genotypes showed differences in traits like flowering time, plant height, ear height, grain yield, harvest moisture response, and protein content under Black Sea conditions in Türkiye. Most compositional and mineral traits remained stable among the materials. P2088 had higher grain yield, KALUMET was later flowering and taller, and DKC6680 had higher protein. These results suggest regional performance differences and potential for further testing. However, due to limited genotypes and specific conditions, more extensive testing is necessary before cultivar recommendations.

## Data Availability

The raw data supporting the conclusions of this article will be made available by the author, without undue reservation.
