# Supplementary material for: Agronomic performance and grain quality assessment of selected maize genotypes in the Black Sea Region of Türkiye
Source: Front Plant Sci. 2026 Aug 6;17:1880606. doi: 10.3389/fpls.2026.1880606 (PMC13489869; doi:10.3389/fpls.2026.1880606)
Supplement: Supplementary file 1 [file Table1.docx]

**Supplementary Table S1.** Description of the six maize genotypes evaluated under Black Sea ecological conditions, including kernel type, source/company or institute, country of origin, maturity group/FAO group, and introduction status.

| **Genotype** | **Type** | **Source / company / institute** | **Country or origin** | **Maturity group / FAO group** | **Introduction status** |
| --- | --- | --- | --- | --- | --- |
| HAV774 | Dent | UPL ZIRAAT VE KIMYA SAN. VE TIC. LTD. STI. | Argentina | 720 | Introduced |
| HAV911 | Dent | UPL ZIRAAT VE KIMYA SAN. VE TIC. LTD. STI. | Argentina | 720 | Introduced |
| HAV923 | Dent | UPL ZIRAAT VE KIMYA SAN. VE TIC. LTD. STI. | Argentina | 700 | Introduced |
| KALUMET | Dent | KWS- Türkiye | Türkiye | 700 | Registered |
| P2088 | Dent | Pioneer- Türkiye | Türkiye | 700 | Registered |
| DKC6680 | Dent | Bayer Türkiye | Türkiye | 700 | Registered |
